# Enhancing nickel stress tolerance in Micro-Tom tomatoes through biopriming with *Paraburkholderia phytofirmans* PsJN: insights into growth and physiological responses

**DOI:** 10.3389/fmicb.2025.1561924

**Published:** 2025-03-13

**Authors:** Mujo Hasanović, Adaleta Durmić-Pašić, Erna Karalija

**Affiliations:** ^1^Institute for Genetic Engineering and Biotechnology, University of Sarajevo, Sarajevo, Bosnia and Herzegovina; ^2^University of Sarajevo, Faculty of Science, Department of Biology, Laboratory for Plant Physiology, Sarajevo, Bosnia and Herzegovina

**Keywords:** biopriming, *Solanum lycopersicum*, *Paraburkholderia phytofirmans PsJN*, nickel stress, physiological response

## Abstract

**Introduction:**

The strategic utilization of plant growth-promoting (PGP) rhizospheric bacteria is a sustainable approach to mitigating the negative effects of anthropogenic activities and excessive nickel (Ni) accumulation in plants. Given that the specific effects of symbiotic interactions depend on the direct relationship between the plant species, bacterial strain, and heavy metals (HMs), this study aimed to investigate the effects of *Paraburkholderia phytofirmans* PsJN seed priming on Ni tolerance in adult Micro-Tom tomato plants (*Solanum lycopersicum L*.).

**Methods:**

Sterilized Micro-Tom seeds were bioprimed with P. phytofirmans PsJN for 24 hours and then sown into the soil. Non-primed, imbibed seeds were used as a control. After 10 days, the seedlings were transferred to a Hoagland nutrient solution. Chronic (10 μM Ni) and acute (50 μM Ni) stress conditions were induced by supplementing the Hoagland solution with Ni salt. The experiment lasted approximately 75 days, covering the complete life cycle of the plants. Various physiological and biochemical parameters were analyzed.

**Results:**

Significant differences (*p* < 0.05) were observed between non-primed and bioprimed tomato plants in terms of fruit yield. Bioprimed tomatoes exhibited higher resilience to Ni stress, particularly under acute stress conditions. Non-primed tomatoes treated with 50 μM Ni showed statistically lower concentrations of chlorophyll a and total chlorophylls compared to bioprimed tomatoes. Moreover, proline content was generally lower and more stable in bioprimed plants, indicating reduced oxidative stress.The activity of antioxidant enzymes exhibited distinct patterns between nonprimed and bioprimed tomatoes.

**Conclusion:**

The findings suggest that biopriming with *P. phytofirmans* PsJN enhances Micro-Tom tomato resilience and growth under Ni stress. This technique appears to mitigate Ni-induced stress effects, particularly at higher Ni concentrations, making it a promising strategy for improving tomato performance in Ni-contaminated environments. Future studies should explore the underlying molecular mechanisms and field applications of this biopriming approach.

## 1 Introduction

The growing challenge of heavy metal (HM) contamination in agricultural soils poses a significant threat to sustainable crop production and food security. Although naturally occurring in soil, Nickel (Ni) became a prevalent heavy metal pollutant through various industrial activities, fossil fuel and coal combustion, mining, and the excessive use of fertilizers and pesticides. Elevated concentrations of Ni in soil can interfere with essential biological processes, leading to toxic effects on overall plant development (Schaumlöffel, 2012).

Ni toxicity disrupts plant physiological and biochemical processes, affecting chlorophyll synthesis, nutrient uptake, and overall growth performance (Hasanuzzaman and Fotopoulos, 2019; Chin et al., 2022; Mustafa et al., 2023; Geetha et al., 2023). Chlorosis and leaf necrosis are common occurrences (Hassan et al., 2019; Jahan et al., 2020), while optimal plant development under high Ni concentrations is often disrupted by reactive oxygen species (ROS) (Rizwan et al., 2018). Consequently, developing sustainable strategies to mitigate Ni-induced stress in crops is imperative for maintaining agricultural productivity.

Plant growth-promoting rhizobacteria (PGPR) have emerged as eco-friendly tools in mitigating abiotic stresses, including heavy metal toxicity. These rhizobacteria enhance plant tolerance through diverse mechanisms, such as improving nutrient availability, modulating hormonal responses, and boosting antioxidant defenses (Singh et al., 2024; Geetha et al., 2023; Chin et al., 2022). Among these, biopriming, a seed treatment technique involving the inoculation of seeds with beneficial microbes, has demonstrated promise in augmenting plant resilience against various stressors. Specifically, biopriming has been reported to enhance germination rates, seedling vigor, and tolerance to HMs by modulating physiological and molecular pathways (Hasanuzzaman and Fotopoulos, 2019; Chin et al., 2022; Mustafa et al., 2023).

*Paraburkholderia phytofirmans* PsJN is a versatile PGPR known for its ability to promote plant growth and alleviate stress under challenging environmental conditions (Esmaeel et al., 2018). Studies have shown its effectiveness in enhancing plant tolerance to heavy metals through mechanisms such as reduced oxidative damage and improved nutrient acquisition (Singh et al., 2024; Geetha et al., 2023). Despite these advancements, limited research has explored the use of *P. phytofirmans* PsJN in alleviating Ni stress, particularly in crops like tomatoes, which are economically and nutritionally important. The Micro-Tom tomato (*Solanum lycopersicum* L.), a well-established model plant in stress physiology studies, offers a unique system to investigate the effects of biopriming on HM tolerance.

This study aims to elucidate the role of biopriming with *P. phytofirmans* PsJN in enhancing Ni stress tolerance in Micro-Tom tomatoes. By evaluating key growth parameters, physiological responses, and biochemical markers, we aim to uncover the underlying mechanisms through which biopriming improves resilience to both chronic and acute Ni stress. Our findings will contribute to the growing body of knowledge on sustainable agricultural practices and the potential application of PGPRs in mitigating environmental challenges.

## 2 Materials and methods

### 2.1 Preparation of *Paraburkholderia phytofirmans* PsJN strain for biopriming

For the preparation of the bacterial inoculum, *Paraburkholderia phytofirmans* PsJN was transferred from a Lauria-Bertani (LB) agar plate into liquid LB medium for overnight incubation. The solid LB medium contained in 1 L: 10 g of tryptone, 5 g of yeast extract, 10 g of NaCl, and 15 g of agar (pH = 7). After incubation at 28°C for 24 h, the cultures exhibiting visible growth were harvested by centrifugation at 4000 rpm for 15 min. After centrifugation, the supernatant was carefully removed, and the bacterial pellet was retained for seed priming. Sterile distilled H_2_O was added to the pellet up to a volume of 50 mL, preparing the medium for priming.

### 2.2 *Solanum lycopersicum* “Micro-Tom” seed sterilization and priming

The tomato seeds “Micro-Tom,” *Solanum lycopersicum* (Aromagärtnerei Deaflora, Germany) were transferred into the priming medium. To acquire the same level of imbibition, the control seeds without bacteria were placed into a tube and filled with sterile distilled H_2_O up to a volume of 50 mL. Both tubes were incubated for 24 h at a temperature of 4°C. After incubation, the seeds were sterilized in 10% Ca-hypochlorite and then rinsed in sterile distilled H_2_O three to four times. After drying on filter paper, the seeds were sown in germination soil (Potgrond H) and transferred to a growth chamber (Phytotron System Instruments; 23°C; 70% humidity, 16 h photoperiod).

To remove soil residues, 15-day-old seedlings were washed in water and transferred to hydroponic containers with Hoagland solution. The Hoagland solution contained in 1 L: 1.5 mL 1 M KNO_3_; 1 mL 1 M Ca (NO_3_) x 4H_2_O; 0.5 mL 1 M NH_4_H_2_PO_4_; 0.25 mL 1 M MgSO_4_ x 7H_2_O; 0.5 mL micronutrients [KCl 0.075 g/L; H_3_BO_3_ 1.59 g/L; MnSO_4_ 0.34 g/L; ZnSO_4_ x 7H_2_O 0.58 g/L; CuSO_4_ x 5H_2_O 0.025 g/L; (NH_4_)_6_Mo_7_O_24_ x 4H_2_O 0.123 g/L; Fe-Na EDTA 0.5 mL]. The plants were left in the Hoagland solution for 7 to 15 days to acclimate to the hydroponic conditions. Total of 15 plants per treatment were used.

### 2.3 Ni as a source of acute and chronic stress

For acute stress, nickel (Ni) in the form of nickel (II) sulfate (NiSO_4_) at a concentration of 50 μM was applied, while for chronic stress, a lower concentration of 10 μM was used in the Hoagland medium. The control group of non-primed and bioprimed seedlings were grown under the same conditions in Hoagland medium without any nickel supplementation. In the acute stress treatment, seedlings were exposed to the Ni solution for a period of 3 weeks, after which the solution was replaced with fresh Hoagland medium without Ni to allow for recovery. This approach was designed to simulate a short-term, high-intensity stress event. In contrast, chronic stress was induced by maintaining a continuous exposure to 10 μM of nickel in the Hoagland medium throughout the entire duration of the experiment. This method replicated long-term, low-level exposure to nickel, as might occur in contaminated environments over extended periods.

Non-primed seeds without Ni are marked as NP 0, while seeds with 10 μM and 50 μM Ni are labeled as NP 10 and NP 50. Bioprimed seeds without Ni are marked BP 0, and those with 10 μM and 50 μM Ni are marked as BP 10 and BP 50, respectively.

### 2.4 Phenotypic analysis and morphometry

During plant growth, phenotypic parameters were monitored: visual symptoms of Ni toxicity (such as chlorosis and/or necrosis), flowering period and fruiting period. The fresh and dry mass of roots, shoots, and fruits were measured. At the end of the experiment (approximately 75 days) fruits of different maturity were selected: green fruits (10–20 days old) and red fruits (40–45 days old). For morphometric analysis the software ImageJ 1.53 k was used (Schneider et al., 2012).

### 2.5 Dry weight and free water content

The effect of Ni on plant fresh and dry weight was evaluated for shoots and roots of primed and non-primed plants under control or Ni spiked conditions. Three representative shoots for each treatment were selected, roots were washed thoroughly to eliminate Hoagland residues and blotted with filter paper to remove excess water. The root and the shoots were separated using a scalpel and the fresh mass was measured, respectively. The samples were subsequently placed in the oven over night at 60°C, after which the dry mass was recorded. The dry weight was expressed in gDW/plant part. The water content was calculated according to the difference between fresh and dry weight and calculated with the following formula:


$$FWC=\frac{FW-DW}{FW}x\;100$$

Where *FWC* represent free water content (as a percentage), *FW* is defined as fresh weight of the plant sample while *DW* refers to the dry weight of the plant sample (Ievinsh, 2023).

### 2.6 Preparation of plant extracts

To extract pigments, a 100 mg sample of air-dried leaves was ground into a fine powder and extracted with 100% acetone. After separating the acetone extracts, the samples were vacuum-dried and subsequently extracted with 80% (v/v) ethanol. This procedure was also performed on other plant tissues. After sonication at 30°C for 15 min, the mixture was centrifuged for 10 min at 2000 rpm. Upon separation of pellet from the supernatant, the extraction procedure was carried out twice. The analysis of soluble sugars and proline was performed using the combined supernatants.

### 2.7 Analysis of plant pigments

The concentration of photosynthetic pigments was determined using a spectrophotometer (Lambda 25, Perkin-Elmer) from 100% acetone extract at the following absorbance wavelengths: 661.6 nm for chlorophyll a (Chl a), 644.8 nm for chlorophyll b (Chl b), and 455.8 nm for carotenoids (Car). The leaves were kept at room temperature and protected from light to preserve the chlorophyll content, as this method retains more chlorophyll compared to other drying techniques (Kumar et al., 2014). After centrifuging at 1500 rpm for 15 min, the supernatant was transferred to a cuvette. For the blank probe, we used 100% acetone solution. The quantification of chlorophylls and carotenoids was conducted according to Lichtenthaler (1987) and expressed in mg/g of dry tissue. The pigment concentrations were calculated using the following formulas:


$$Chl\;a\;\left(\mathrm{\mu g}/\mathrm{mL}\right)=11.24\;x\;A661.6-2.04\;x\;A644.8\text{.}$$

$$Chl\;b\;\left(\mathrm{\mu g}/\mathrm{mL}\right)=20.13\;x\;A644.8+18.09\;x\;A661.6$$

$$\frac{\mathrm{Car}\;\left(\mathrm{\mu g}/\mathrm{mL}\right)=\left(1000\;x\;A455.8-1.9\;x\;Chl\;a-63.14\;x\;Chl\;b\right)}{214}$$

The letter A represents the absorbance at different wavelengths. The molar absorption coefficients for 100% acetone with a 1-cm path length are given as 11.24, 20.13, 2.04, 18.09, 1.9 and 63.14.


$$C=\frac{C1\mathrm{xVxR}}{M}$$

In this context, *C* denotes the pigment concentration in the dry material (mg/g), while *C1* refers to the pigment concentration in the acetone extract solution (mg/mL). *V* represents the total volume of the plant extract, and *R* is the dilution factor. Lastly, *M* corresponds to the mass of the plant material (gDW).

### 2.8 Analysis of proline content

For the analysis of proline content, a modified method by Carillo et al. (2008) was employed using an 80% ethanol extract. In each 15 mL tube, 1 mL of the extract was combined with 5 mL of a solution containing 1% ninhydrin. 60% acetic acid, and 20% ethanol. The absorbance was measured at 520 nm, and L-proline was used to prepare the calibration curve. The proline content was expressed using the following formula:


$$\begin{array}{l} \mathrm{Proline}\;\left(\mathrm{in}\frac{\mathrm{nmol}}{\mathrm{mg}\;DW}\;\mathrm{or in}\;\frac{\mathrm{\mu mol}}{\mathrm{mgDW}}\right)\\ =\left(\frac{\mathrm{Absextract}-\mathrm{Absblank}}{\mathrm{slope}}\right)x\;\left(\frac{\mathrm{Volextract}\;}{\mathrm{Volaliquot}}\right)x\frac{1}{DW} \end{array}$$

The absorbance of the extract is denoted as Abs*_extract_*, while the blank probe absorbance is indicated as Abs*_blank_*. The slope, defined as absorbance/nmol, is calculated through linear regression. Vol*_extract_* refers to the volume of the ethanol extract, and Vol*_aliquot_* represents the volume of extract used in the reaction. The proline content is expressed as mg/gDW.

### 2.9 Analysis of protein content

Total protein content was measured using the Bradford (1976) method, with bovine serum albumin (BSA) as the protein standard. The standard curve for calculating the content of protein was created using serial dilutions of BSA. The absorbance was measured at 595 nm and protein content was presented as mg/gFW.

### 2.10 Analysis of soluble sugars

Soluble sugars were extracted with 80% ethanol and quantified spectrophotometrically using the anthrone method, following a modified protocol based on Marshall (1986). The anthrone reagent was prepared by dissolving 1 g of anthrone in 500 mL of 72% sulfuric acid. In each 15 mL tube, 1 mL of the 80% ethanol plant extract and 5 mL of anthrone reagent were added. Some samples were further diluted with 80% ethanol before analysis. The samples were incubated for 11 min at 100°C in a water bath and rapidly cooled on ice to stop the reaction. Absorbance was measured at 630 nm, and a mixture of 80% ethanol and anthrone reagent was used as the blank probe.

### 2.11 Determination of antioxidant enzymes

The enzymatic activity of guaiacol peroxidase (POD) was determined according to a modified method by Angelini et al. (1990) for plant extracts diluted 500 times in 0.1 M phosphate buffer (pH = 7) containing 5 mM guaiacol and 0.1 M H_2_O_2_. The enzymatic activity was determined by measuring the change in absorbance after an interval of 120 s, relative to the protein content. The results were presented using the extinction coefficient of the guaiacol dehydrogenation product tetraguaiacol (*ε* = 26.6 mM − 1 cm − 1) and expressed in enzyme units per milligram of protein. One unit of the enzyme represents the amount of enzyme required to oxidize 1 μM of guaiacol per minute.

A modified protocol by Nakano and Asada (1981) was used for the analysis of ascorbate peroxidase (APX) by measuring the decrease in absorbance at 290 nm over 1 min. The reaction mixture consisted of 50 mM phosphate buffer (pH = 7), 0.1 mM EDTA, 0.5 mM ascorbic acid, and 20 mM H_2_O_2_. The reaction was initiated by adding the enzyme extract, and the activity was expressed as the rate of ascorbate oxidation (*ε* = 0.74 mM^−1^ cm^−1^), measured in units of APX. One unit of APX was defined as the amount of enzyme required to oxidize 1 μM of ascorbic acid per minute.

The activity of catalase (CAT) was evaluated through the formation of a stable complex between H_2_O_2_ and (NH₄)₆Mo₇O₂₄, according to a modified method by Góth (1991). The enzyme extract was added to the reaction mixture containing 65 mM H_2_O_2_ in 60 mM phosphate buffer (pH = 7.4). After incubation for 4 min at 25°C, the reaction was stopped by adding 32.4 mM (NH₄)₆Mo₇O₂₄, and the absorbance was measured at 405 nm. Catalase activity was calculated as the rate of H_2_O_2_ reduction (ε = 39.4 mM − 1 cm − 1) and expressed in units of CAT per milligram of protein. One unit of CAT was defined as the amount of enzyme required to remove 1 mM of H_2_O_2_ per minute.

### 2.12 Statistical analysis

The statistical analysis involved data from morphological, biochemical and physiological parameters, with measurements conducted in triplicate. Results were expressed as mean values with standard deviation (±SD). The Shapiro–Wilk test was used to assess the normality of the data distribution. For parametric data, one-way ANOVA was applied to analyze variance, followed by the Tukey (HSD) test for *post hoc* analysis. For data that did not meet the assumptions of normality, Kruskal-Wallis non-parametric test was used (GraphPad Prism version 9.0.2). A significance level of *p* < 0.05 was set for the analysis. Different letters indicate statistical differences within NP and BP treatments while asterisk indicate statistical differences between NP and BP groups within individual Ni concentration (*p* < 0.05).

## 3 Results

### 3.1 Biopriming ameliorates growth-stunting effects of Ni

**Figure 1:**
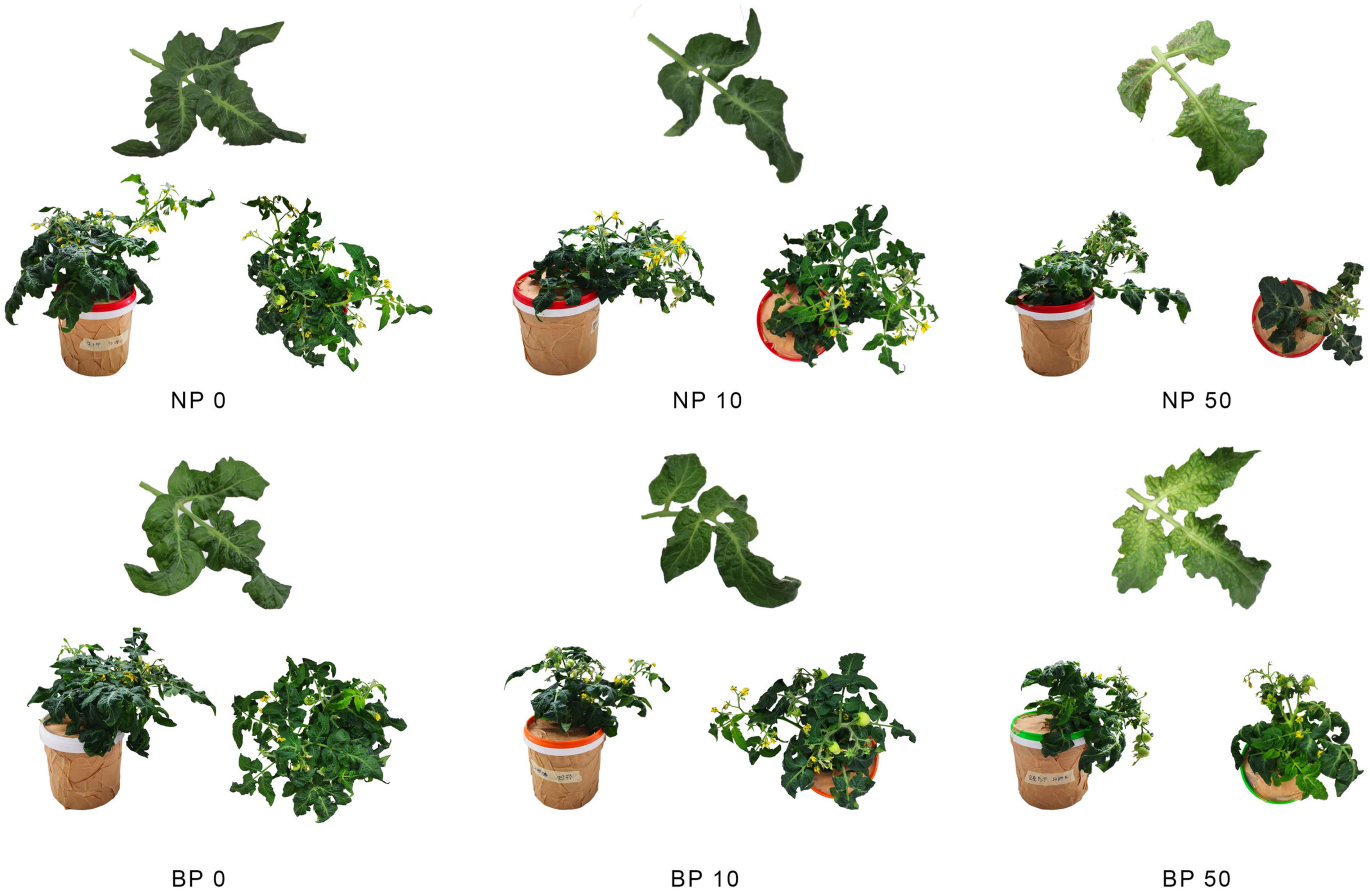
Visual representation of non-primed (NP) and bioprimed (BP) plants grown under different Ni concentrations (0, 10 and 50 μM).

**Figure 2:**
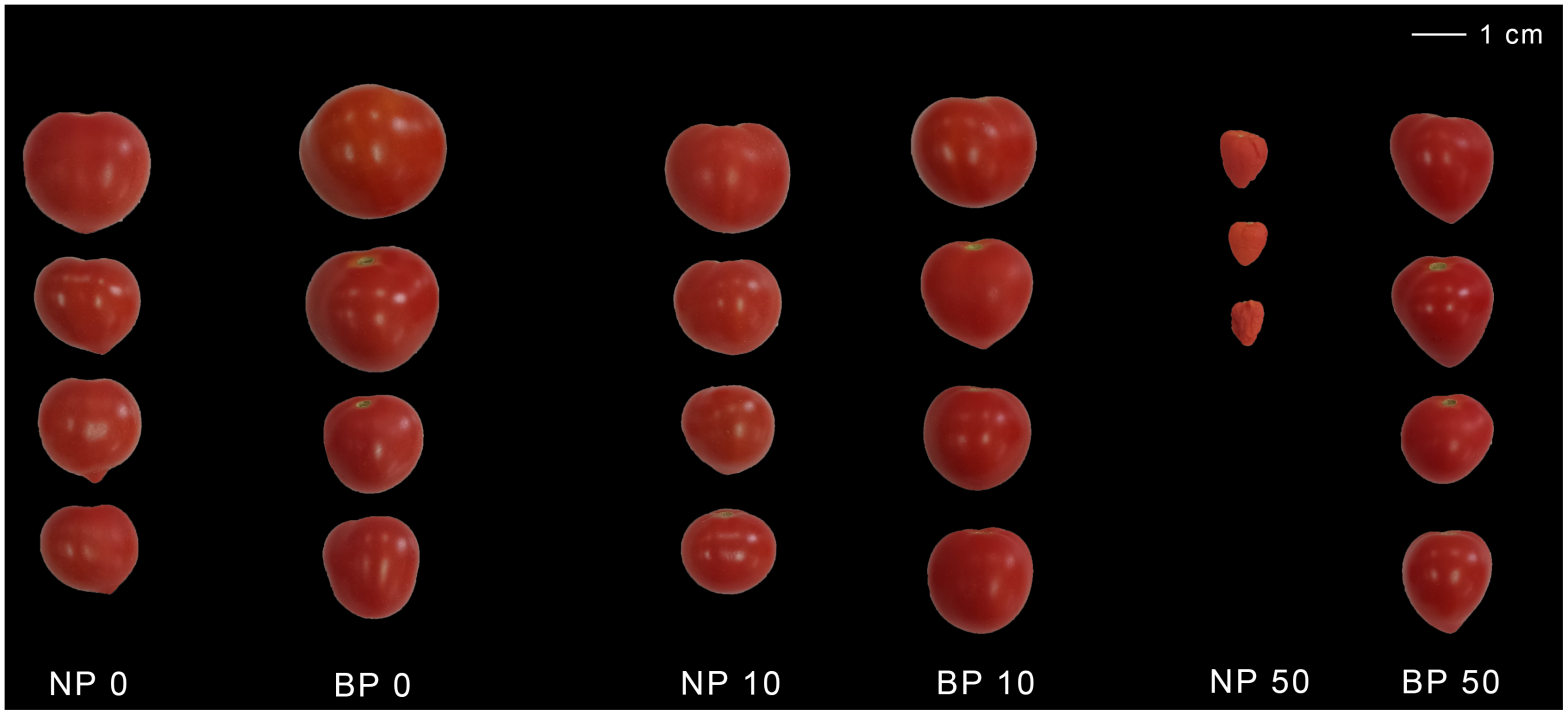
Mature fruit size of non-primed (NP) and bioprimed (BP) plants under different Ni concentrations (0, 10 and 50 μM).

Non-primed Micro-Tom plants exposed to acute and chronic stress exhibited growth inhibition and the disruption of photosynthesis and transpiration, which are known companions of Ni toxicity (Sreekanth et al., 2013). The key signs of toxicity; including chlorosis, leaf necrosis, and stunted growth were more pronounced in groups exposed to acute Ni stress (50 μM Ni). Typical examples of the visual symptoms of nickel toxicity in NP and BP plants are shown in Figure 1. Most apparent differences in mature fruit size are observed between NP 50 and BP 50. NP 50 plants in recovery after acute stress did complete their phenological cycle but developed fruits were small, without seeds, dry and showed signs of senescence, while BP 50 plants had normal fruit size, but smaller number of seeds compared to BP0 (Figure 2). Chronic stress induced acceleration of phenological cycle, and plants flowered, fruited earlier, and achieved fruit maturity 15 days before control, while in case of acute stress plant growth was stunted and phenological cycle was delayed 15 days compared to control plants.

### 3.2 Biopriming increases tomato tolerance to Ni stress and positively affects its growth

**Figure 3:**
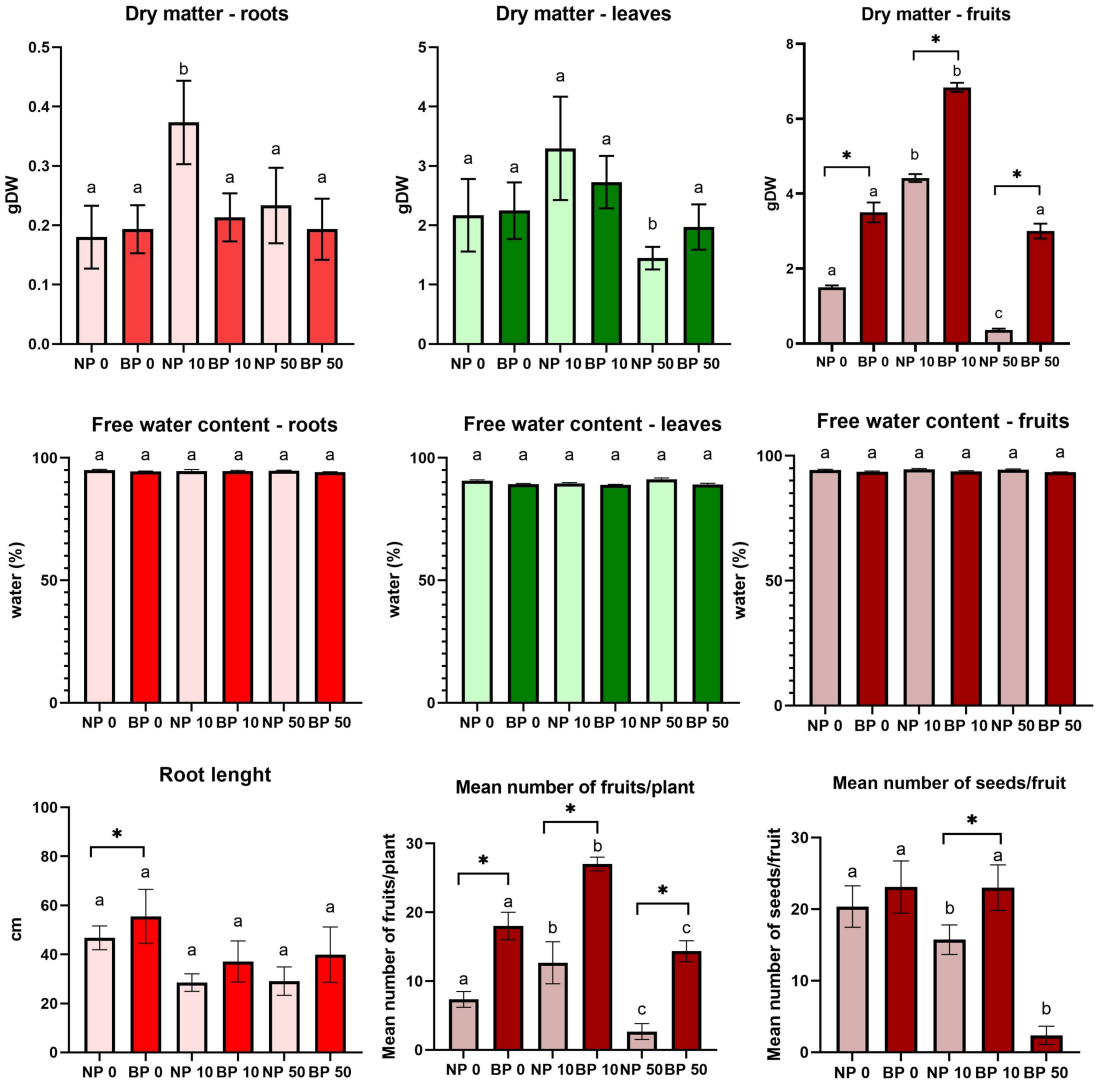
Root length, dry matter and free water content in roots, leaves and fruits, along with the mean number of fruits per plant and seeds per fruits of non-primed (NP) and bioprimed (BP) plants under different Ni concentrations (0, 10 and 50 μM). Different letters indicate statistical differences within NP and BP treatments while asterisk indicate statistical differences between NP and BP groups within individual Ni concentration (*p* < 0.05). NP 50 plants were excluded from the calculation of the mean seed number per fruit as they produced seedless fruit.

Regardless of treatment and type of Ni exposure in NP plants, the dry matter of roots and leaves, as well as root length in NP plants, were affected. However, no significant differences were observed, except for the root dry matter in NP 10. Similar trend was observed for BP as well, but the stress effects are alleviated compared to NP plants (Figure 3). In correlation to the accelerated phenological cycle, plants under chronic stress showed a higher number of produced fruits for NP and BP plants. Under Ni stress NP plants produced fruits with less seed, with fruits of acute stressed plants being completely senescing and seedless.

### 3.3 Biopriming maintains photosynthetic pigment levels in plants under nickel stress

**Figure 4:**
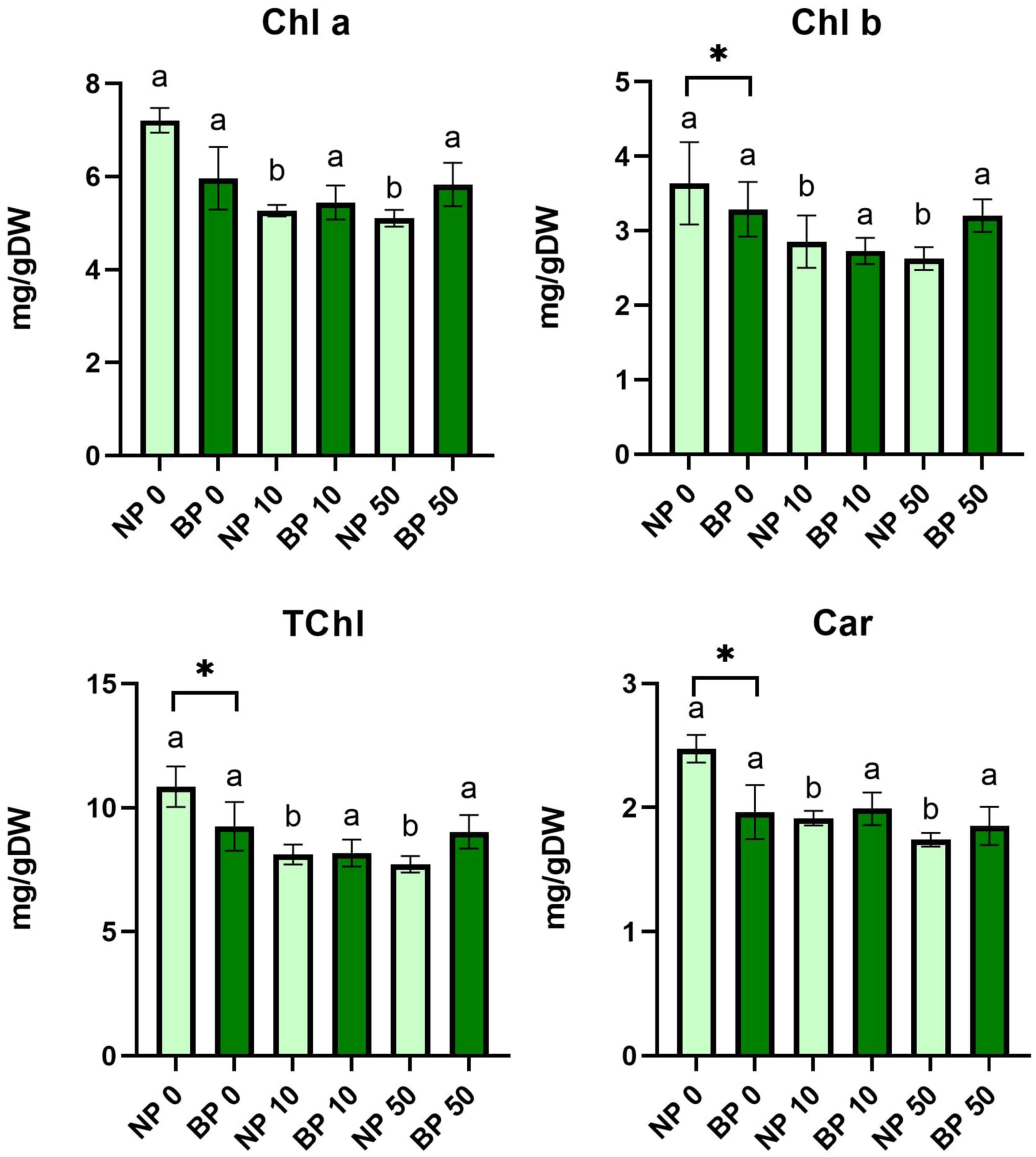
The content of photosynthetic pigments and carotenoids between non-primed (NP) and bioprimed (BP) plant groups under different exposure to Ni (0, 10 and 50 μM). Different letters indicate statistical differences within NP and BP treatments while asterisk indicate statistical differences between NP and BP groups within individual Ni concentration (*p* < 0.05).

The statistical analysis of Chl a, Chl b, total chlorophyll (TChl) and Car revealed a significant decrease in these parameters in NP plants under both acute and chronic Ni stress compared to control NP 0. On the other hand, pigment content in BP plants was not significantly affected, showing no statistical differences between different types of Ni exposure. Additionally, significant differences were also detected between NP 0 and BP 0 in overall concentration of Chl b, TChl and Car (Figure 4).

### 3.4 Proline and sugar content changes under Ni stress in non-primed and bioprimed plants, respectively

**Figure 5:**
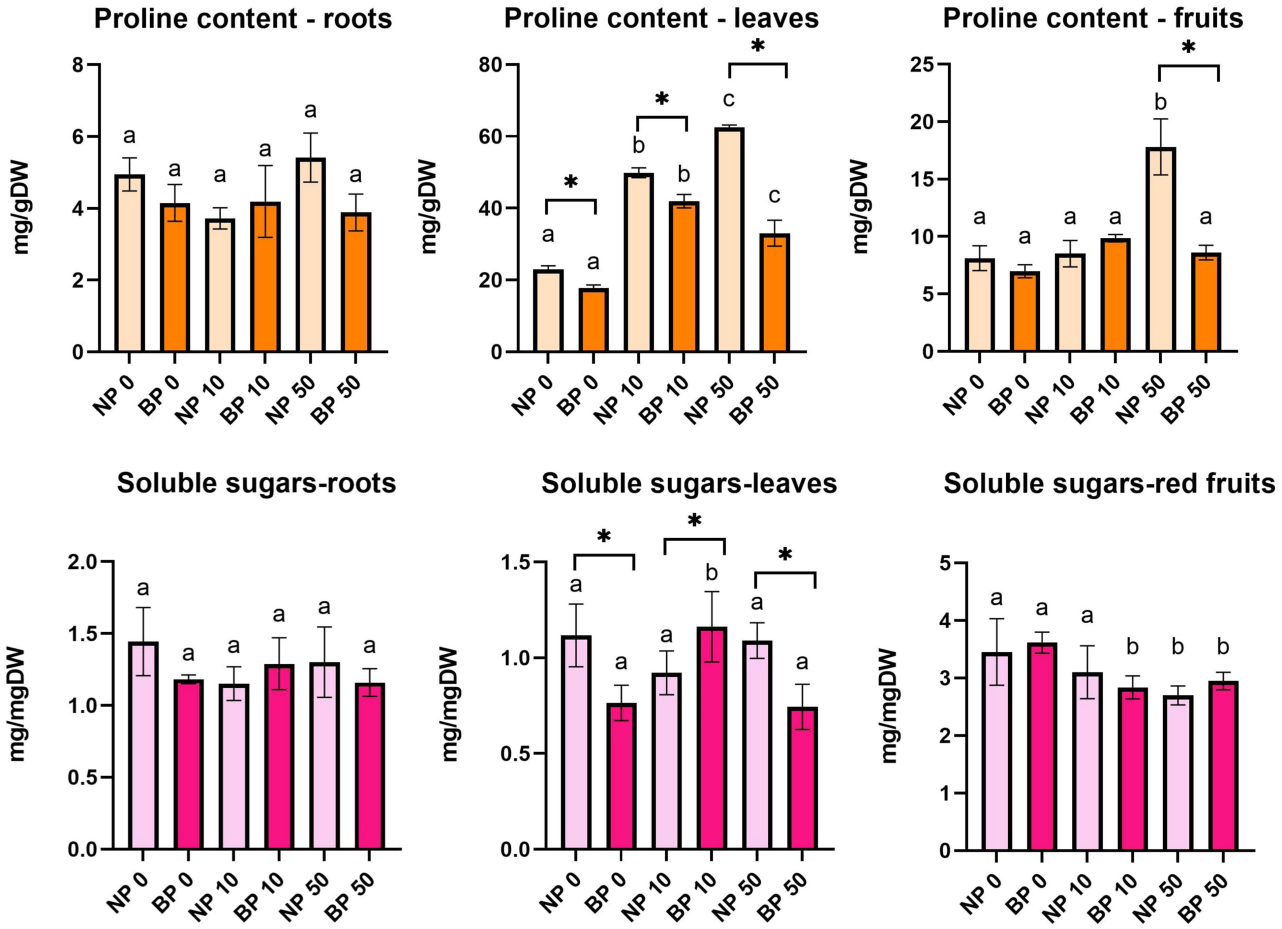
The content of proline and soluble sugars in leaves, roots and red fruits of non-primed (NP) and bioprimed (BP) plant under different exposure to Ni (0, 10 and 50 μM). Different letters indicate statistical differences within NP and BP treatments while asterisk indicate statistical differences between NP and BP groups within individual Ni concentration (*p* < 0.05).

The content of proline varied among treatments and groups (Figure 5). Proline content in leaves was significantly higher in NP 10 and NP 50 compared to NP 0. Similar trend was observed for BP treatments compared to BP 0, although BP 10 showed significantly higher proline content compared to BP 0 and BP 50. In general, BP plants had lower proline content compared to NP plants. No significant difference in proline content was observed in roots.

NP 50 showed significantly higher proline content in fruits compared to NP 0 and NP 10, while no statistical difference was observed among BP treatments. NP 50 also showed significantly higher proline concentration in fruits compared to BP 50.

While content of soluble sugars did not show any differences in roots, soluble sugars in leaves and red fruits varied (Figure 5). BP 10 showed significantly higher soluble sugar content in leaves compared to BP 0 and BP 50, while no statistical difference was observed within NP group under different Ni concentrations. A significantly higher concentration of soluble sugars in leaves was observed in NP 0 and NP 50 compared to BP 0 and BP 50, respectively, but BP 10 had higher concentration compared to NP 10.

The content of soluble sugars in red fruits did not vary between NP and BP groups within individual Ni concentration. However, statistical differences were observed within both NP and BP groups. BP 0 revealed higher soluble sugar content compared to BP 10 and BP 50 while in NP group only NP 50 showed statistically lower concentration of soluble sugars compared to NP 0 and NP 10.

### 3.5 Protein content varied between non-primed and bioprimed plants

Protein content in leaves did not reveal significant differences among NP treatments and the concentration was relatively stable. BP 0 showed significantly higher protein content in leaves compared to BP 10 and BP 50. In addition, the content of protein in BP 0 was significantly higher compared to NP 0.

Protein content in roots was generally higher in all BP treatments compared to NP. Significantly higher protein content was observed between BP 0 and NP 0 and BP 50 and NP 50. In comparison to BP 0 and BP 50, BP 10 showed statistically lower concentration of protein content in roots.

**Figure 6:**
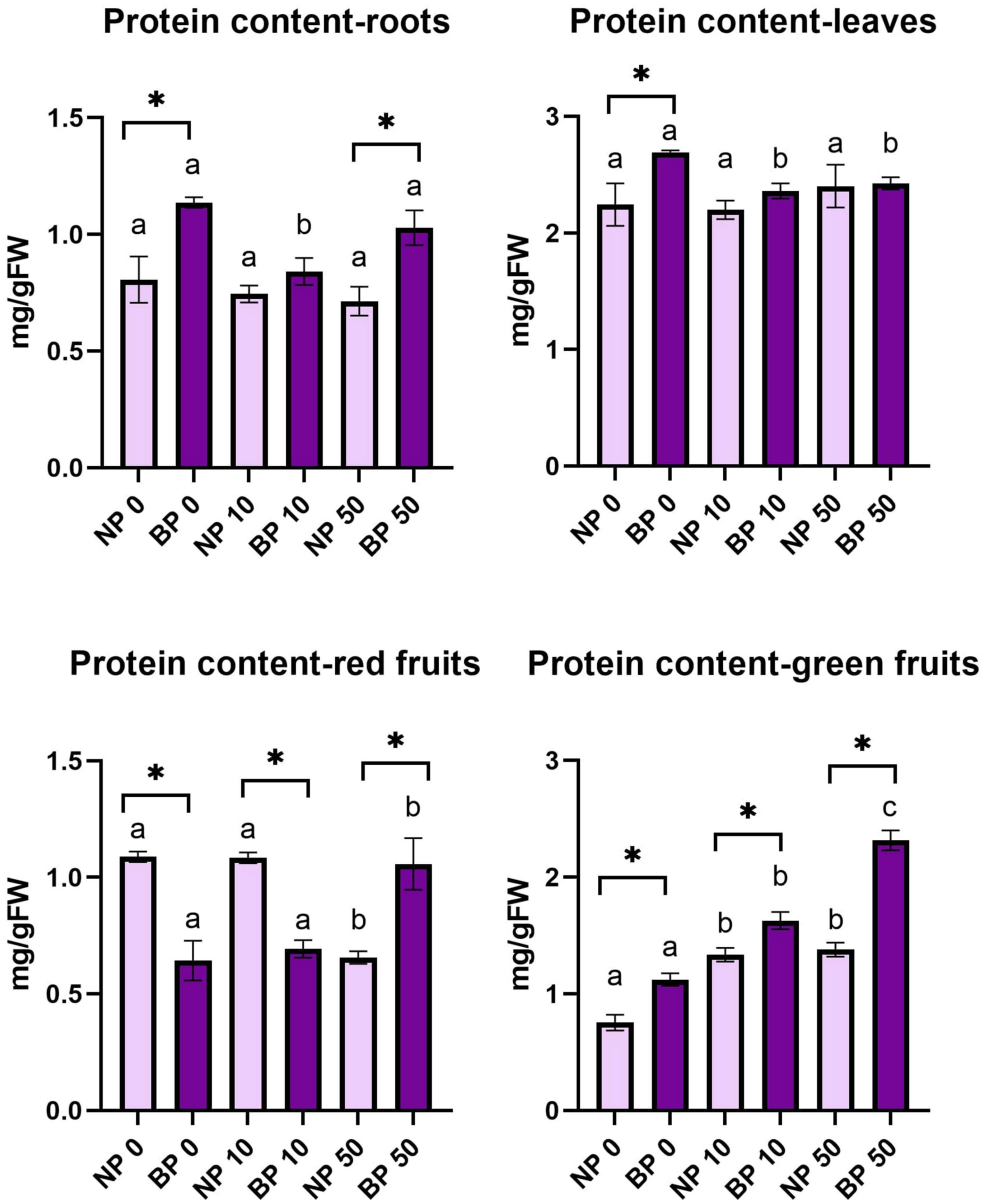
Protein content in leaves, roots, red and green fruits of non-primed (NP) and bioprimed (BP) plant under different exposure to Ni (0, 10 and 50 μM). Different letters indicate statistical differences within NP and BP treatments while asterisk indicate statistical differences between NP and BP groups within individual Ni concentration (*p* < 0.05).

In red fruits, the protein content showed variation among all the combinations of priming and Ni treatment. Within NP treatments, NP 50 showed significantly lower protein concentration compared to NP 0 and NP 10. In contrast, BP 50 showed significantly higher protein content compared to BP 0 and BP 10. Among groups, we observed significant differences in all Ni treatments. Protein content in green fruits was significantly higher in BP in all observed treatments compared to NP. Within groups, significant differences were also observed among all BP treatments while NP 0 had statistically lower protein content compared to NP 10 and NP 50 (Figure 6).

### 3.6 Biopriming enhances antioxidant capacity through increase of catalase activity

**Figure 7:**
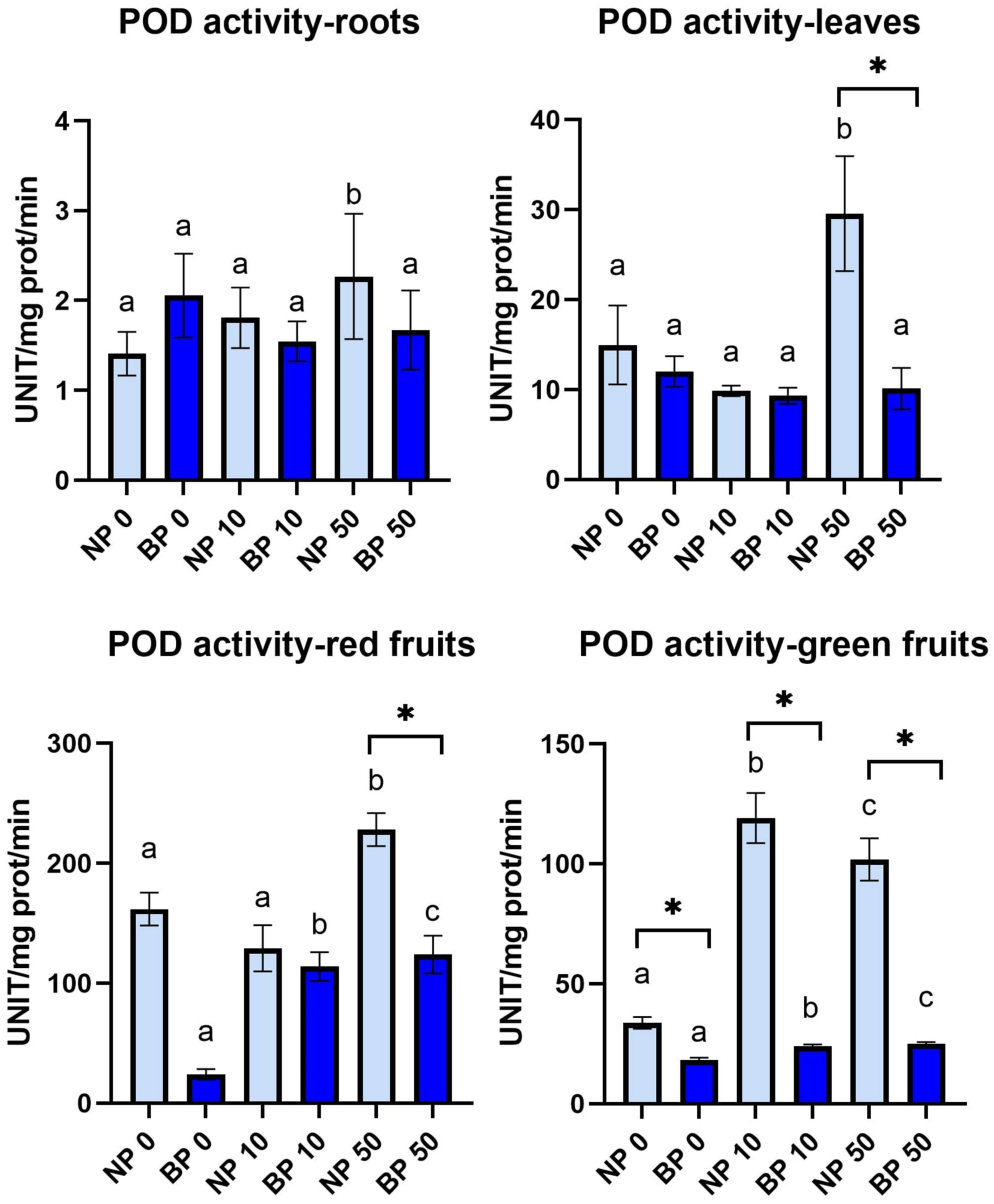
The activity of POD in leaves, roots, red and green fruits of non-primed (NP) and bioprimed (BP) plant under different exposure to Ni (0, 10 and 50 μM). Different letters indicate statistical differences within NP and BP treatments while asterisk indicate statistical differences between NP and BP groups within individual Ni concentration (*p* < 0.05).

POD activity in leaves was relatively stable with no observable significant differences except NP 50 which was statistically higher compared to NP 0 and NP 10. NP 50 also had significantly higher POD activity compared to BP 50. Similar situation was observed in roots with NP 50 just without significant difference compared to BP 50. POD activity in red and green fruits generally showed higher activity in NP treatments compared to BP. Significantly higher POD activity in red fruits was observed in NP 0 and NP 50 compared to BP 0 and BP 50, respectively. Within NP treatments, NP 50 revealed significantly higher POD activity compared to NP 0 and NP 10 while BP 0 showed significantly lower POD activity compared to BP 10 and BP 50. All NP treatments showed significantly higher POD activity in green fruits compared to BP. POD activity was statistically different in all NP treatments while BP treatments did not show any significant differences (Figure 7). APX activity was not detected in any of the tested treatments and groups.

**Figure 8:**
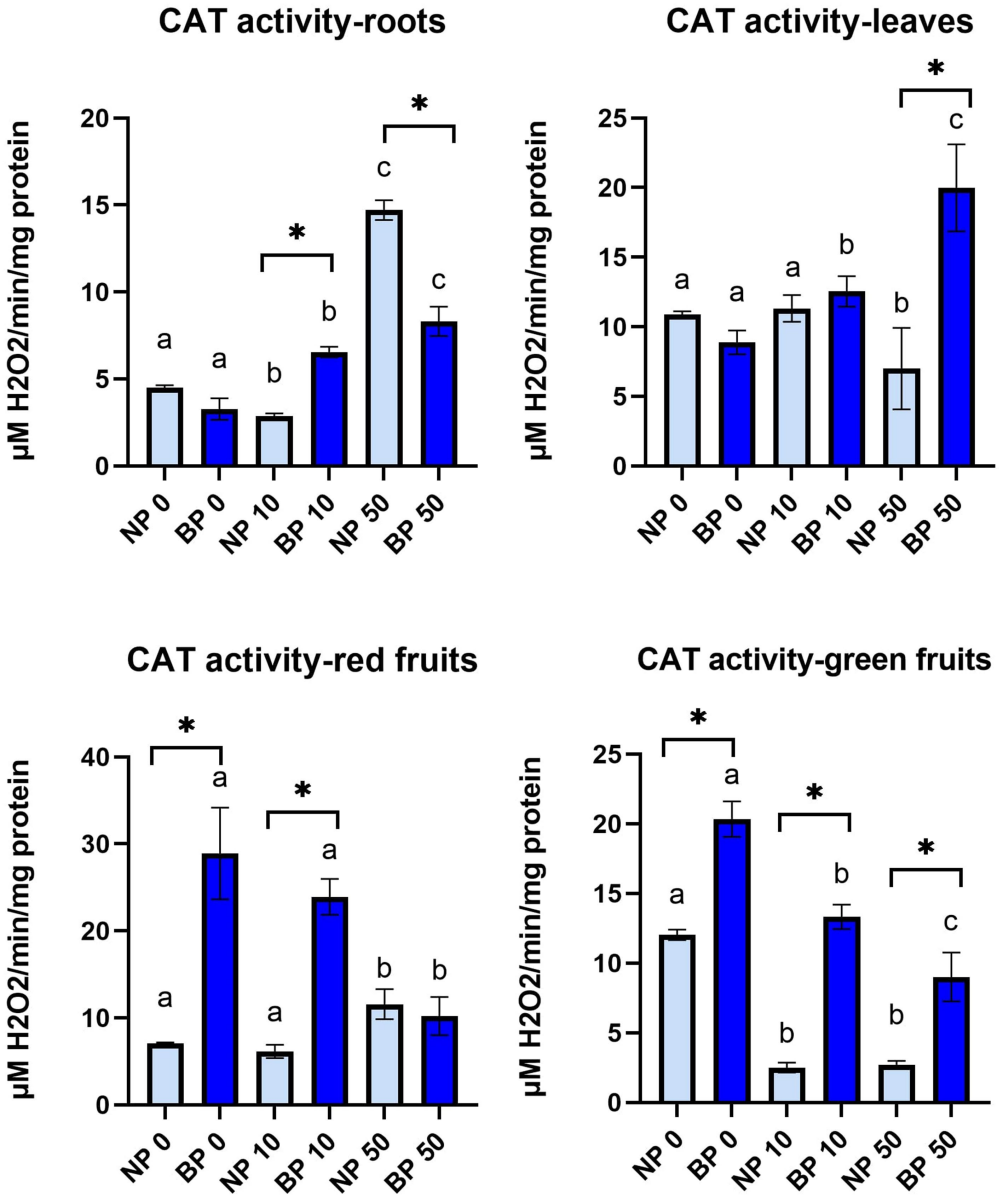
The activity of CAT in leaves, roots, red and green fruits of non-primed (NP) and bioprimed (BP) plant under different exposure to Ni (0, 10 and 50 μM). Different letters indicate statistical differences within NP and BP treatments while asterisk indicate statistical differences between NP and BP groups within individual Ni concentration (*p* < 0.05).

CAT activity generally showed higher activity in BP treatments especially in red and green fruits (Figure 8). In leaves, CAT activity in NP 50 was significantly lower compared to NP 0 and NP 10. On the other hand, all BP treatments revealed significant differences from one another. In group comparison, only BP 50 showed significantly higher CAT activity than NP 50. CAT activity in roots differed among treatments with Ni. Within groups, all treatments showed significant differences in CAT activity with highest in NP 50 and BP 50, while between groups significantly higher CAT activity was observed in BP 10 compared to NP 10 and NP 50 compared to BP 50.

Red fruits of BP 0 and BP 10 revealed significantly higher CAT activity compared to NP 0 and NP 10, respectively. Within groups, both NP and BP treatments with 50 μM showed significant differences. In green fruits, all BP treatments showed significantly higher CAT activity compared to NP. Within groups, NP 0 showed higher CAT activity compared to NP 10 and NP 50 while all BP treatments significantly differed from each other with highest CAT activity in BP 0 and lowest in BP 50 (Figure 8).

## 4 Discussion

### 4.1 Biopriming ameliorates growth-stunting effects of Ni

The toxic accumulation of Ni in agricultural soils can disrupt many key physiological processes, including photosynthesis and transpiration (Sreekanth et al., 2013), and inhibit plant metabolism (Sreekanth et al., 2013; Mustafa et al., 2023), consequently leading to stunted growth and chlorosis, as observed in non-primed (NP) Micro-Tom plants exposed to acute and chronic Ni stress in this study. In non-primed plants acute stress induced more pronounced symptoms, such as chlorosis and necrosis, while chronic stress accelerated the phenological cycle of the plants compared to control. These observations align with previous findings highlighting Ni′s detrimental impact on plant growth, root development, and biomass accumulation (Gupta et al., 2017; Hassan et al., 2019; Roccotiello et al., 2022; Yu et al., 2024). The absence of seeds in NP 50 may be linked to hormonal imbalances involving abscisic acid (ABA), gibberellin (GA), and auxin. ABA’s role in heavy metal stress responses, including seed development inhibition through interactions with GA, has been well-documented (Bücker-Neto et al., 2017; Shu et al., 2018).

Bioprimed (BP) plants demonstrated higher resilience, completing their phenological cycles with reduced signs of stress and improved fruit development compared to NP plants, aligning with previous reports of biopriming enhancing plant stress tolerance (Hasanuzzaman and Fotopoulos, 2019). While BP 50 plants also showed reduced seed numbers, they maintained normal fruit size and overall healthier phenological development. This outcome supports previous studies on the efficacy of *Paraburkholderia phytofirmans* PsJN in mitigating abiotic stresses, including heavy metals, through mechanisms such as enhanced nutrient uptake and hormonal modulation (Naveed et al., 2014; Pinedo et al., 2015).

### 4.2 Biopriming increases tomato tolerance to Ni stress and positively affects growth

The root system of NP plants under Ni stress was significantly inhibited, showing reduced length, water content, and biomass, especially under acute stress conditions. These effects align with findings that elevated Ni concentrations interfere with root function and nutrient absorption (Hassan et al., 2019). In contrast, BP plants exhibited less pronounced reduction, suggesting that biopriming with *P. phytofirmans* PsJN enhances root resilience, where biopriming could contribute to improving root architecture and water-use efficiency (Chin et al., 2022).

As mentioned, chronic Ni stress accelerated the phenological cycle of Micro-Tom plants, leading to earlier flowering and fruiting. Stress-induced flowering, previously described under other abiotic stresses such as cadmium (Wang et al., 2012), prioritizes reproduction over growth as a survival mechanism. While NP 10 exhibited a potential hormetic response, characterized by increased dry matter content under moderate stress, BP 10 plants did not exhibit such pronounced effects, instead maintained better overall growth and fruit quality. Hormesis, a biphasic response to stress, is well-documented in plants and may partially explain the observed trends in non-primed plants (Poschenrieder et al., 2013; Jalal et al., 2020).

#### 4.2.1 Biopriming maintains photosynthetic pigments and reduces stress-induced proline accumulation

Ni stress significantly reduced photosynthetic pigments, including chlorophylls and carotenoids, in NP plants as previously recorded in tomato seedlings exposed to high Ni concentrations (Yu et al., 2024). This reduction correlates with known effects of Ni disrupting chloroplast structures and photosystem II (PSII) functionality, as Ni replaces essential cations like Mg^2+^ and Ca^2+^ at binding sites (Boisvert et al., 2007; Hassan et al., 2019). In contrast, BP plants maintained stable pigment levels across treatments, consistent with previous studies demonstrating the role of *P. phytofirmans* PsJN in enhancing photosynthetic performance under stress (Issa et al., 2018; Nafees et al., 2018).

Proline accumulation, a common response to heavy metal stress, was significantly higher in NP plants under Ni stress. While elevated proline levels are often associated with osmoprotection and ROS quenching (Hayat et al., 2012; Rehman et al., 2021), the higher accumulation observed in NP plants likely indicates increased oxidative stress. In contrast, BP plants exhibited lower proline levels, suggesting that biopriming mitigates stress more effectively (Srivastava et al., 2024), reducing the need for excessive osmoprotectants, thus resulting enhanced growth since less energy is used for osmolyte biosynthesis.

#### 4.2.2 Soluble sugar content and antioxidant enzyme activity

Soluble sugar content varied among treatments, with NP plants generally accumulating higher levels in leaves compared to BP plants. This suggests that NP plants rely more heavily on sugars as a primary stress response due to less efficient antioxidant defenses. BP 10 plants showed significantly higher sugar content in leaves compared to other BP treatments, indicating that moderate Ni stress might enhance metabolic adaptations in bioprimed plants. Heavy metals, including Ni, adversely affect carbohydrate metabolism, which was reflected in reduced sugar content in fruits across treatments (Hashem et al., 2018).

Antioxidant enzyme activity showed distinct patterns between NP and BP plants. POD activity was significantly higher in NP treatments, particularly in fruits, reflecting heightened oxidative stress and ROS proliferation. Conversely, CAT activity was more pronounced in BP plants, supporting the hypothesis that biopriming enhances ROS detoxification by favoring catalase activity, which directly neutralizes H₂O₂ without requiring substrates (Dumanović et al., 2021). These results are consistent with findings that biopriming enhances antioxidant defenses, leading to better stress management (Gianella et al., 2021; Fiodor et al., 2023; Srivastava et al., 2024).

Overall, biopriming with *P. phytofirmans* PsJN enhanced Micro-Tom tomato plants resilience to nickel stress, supporting sustainable agriculture by maintaining productivity in contaminated soils and reducing the need for chemical inputs. Biopriming is known to improve plant tolerance to various abiotic stresses, such as drought and temperature fluctuations, which contributes to climate change resilience (Singh et al., 2023). This can help maintain crop yields and food security under changing environmental conditions. By promoting healthier soil microbiomes and plant growth, biopriming indirectly supports ecosystem services like carbon sequestration, aiding in climate change mitigation efforts.

## 5 Conclusion

Biopriming with *P. phytofirmans* PsJN significantly improved the physiological and morphological performance of Micro-Tom tomatoes under Ni stress. Enhanced pigment stability, reduced proline accumulation, and optimized antioxidant enzyme activity collectively contributed to better growth and yield in BP plants compared to NP. These results underscore the potential of biopriming as a sustainable agricultural practice to mitigate heavy metal stress. However, in polluted soils Ni is frequently associated with Cd, Pb, Co and/or Zn; further studies should examine the effectiveness of biopriming in real-world scenarios marked by co-pollution. In addition, open field trials should be conducted to evaluate the practicality of biopriming applications under diverse environmental conditions. Further studies exploring the hormonal mechanisms, modulation of antioxidant enzyme activities and nutrient uptake pathways involved in biopriming under Ni stress could provide deeper insights into the mechanics of biopriming with *P. phytofirmans* PsJN. Understanding the mechanisms would enable targeted application of biopriming to a broader range of crops to enhance their resilience against heavy metal stress.

## Data Availability

The raw data supporting the conclusions of this article will be made available by the authors, without undue reservation.
